# Part and Parcel of the Cardiac Autonomic Nerve System: Unravelling Its Cellular Building Blocks during Development

**DOI:** 10.3390/jcdd3030028

**Published:** 2016-09-12

**Authors:** Anna M. D. Végh, Sjoerd N. Duim, Anke M. Smits, Robert E. Poelmann, Arend D. J. ten Harkel, Marco C. DeRuiter, Marie José Goumans, Monique R. M. Jongbloed

**Affiliations:** 1 Department of Molecular Cell Biology, Leiden University Medical Center, Einthovenweg 20, 2333 ZC Leiden, The Netherlands; a.m.d.vegh@lumc.nl (A.M.D.V.); s.n.duim@lumc.nl (S.N.D.); A.m.smits@lumc.nl (A.M.S.); M.J.Goumans@lumc.nl (M.J.G.); 2 Department of Cardiology, Leiden University Medical Center, Albinusdreef 2, 2333 ZC Leiden, The Netherlands; r.e.poelmann@lumc.nl; 3 Institute of Biology Leiden, Leiden University, Sylviusweg 20, 2311 EZ Leiden, The Netherlands; 4 Department of Pediatric Cardiology, Leiden University Medical Center, Albinusdreef 2, 2333 ZC Leiden, The Netherlands; A.D.J.ten_Harkel@lumc.nl; 5 Department of Anatomy & Embryology, Leiden University Medical Center, Einthovenweg 20, 2333 ZC Leiden, The Netherlands; M.C.de_Ruiter@lumc.nl

**Keywords:** nervous system, cardiac, autonomic, development, innervation, cells, neurotrophic factors, neural crest cells

## Abstract

The autonomic nervous system (cANS) is essential for proper heart function, and complications such as heart failure, arrhythmias and even sudden cardiac death are associated with an altered cANS function. A changed innervation state may underlie (part of) the atrial and ventricular arrhythmias observed after myocardial infarction. In other cardiac diseases, such as congenital heart disease, autonomic dysfunction may be related to disease outcome. This is also the case after heart transplantation, when the heart is denervated. Interest in the origin of the autonomic nerve system has renewed since the role of autonomic function in disease progression was recognized, and some plasticity in autonomic regeneration is evident. As with many pathological processes, autonomic dysfunction based on pathological innervation may be a partial recapitulation of the early development of innervation. As such, insight into the development of cardiac innervation and an understanding of the cellular background contributing to cardiac innervation during different phases of development is required. This review describes the development of the cANS and focuses on the cellular contributions, either directly by delivering cells or indirectly by secretion of necessary factors or cell-derivatives.

## 1. Development of the Heart and Cardiac Innervation

The cardiac autonomic nervous system (cANS) modulates physiological cardiac functions such as heart rate and contraction force. The heart is innervated by nerves from the brain and spinal cord that either stimulate or inhibit these cardiac functions. Under pathological conditions, the density of these nerve fibers in the heart may be altered, leading to either too much activation (hyperinnervation) or too little (hypoinnervation). This (regional) difference in innervation may cause a spatial imbalance in the activation of the heart, leading to cardiac autonomic dysfunction [1]. Cardiac autonomic dysfunction is related to the development and progression of cardiovascular diseases such as myocardial infarction (MI), arrhythmias, hypertension and heart failure [2,3,4,5], and ultimately may lead to sudden cardiac death [6]. In addition, the prognosis of patients with congenital heart disease is negatively influenced by autonomic dysfunction, as well as in other patient groups such as after a cardiac transplantation [5,7,8].

The development of innervation itself and the role it plays during the fetal period may be related to problems with innervation in later life. Pressure overload-induced hypertrophy is accompanied by regional hyperinnervation of sympathetic neurons and, interestingly, these neurons showed a fetal phenotype [1]. Therefore, unravelling the embryonic development of the cANS may help to understand the process behind pathological cardiac hyper- and hypoinnervation and may give insights into new therapeutic avenues.

The development of cardiac autonomic innervation is a complex process that occurs in a temporally and spatially controlled manner. Many cell types are involved in establishing the cANS, either directly by delivering cellular building blocks, or indirectly by secreting factors that induce differentiation, repulsion, or attraction of other cells. This review focuses on cell populations that contribute to the development of the cANS, as well as on related neurotrophic factors and signaling pathways during embryonic and fetal development.

### 1.1. Embryonic Development of the Heart

**Figure 1:**
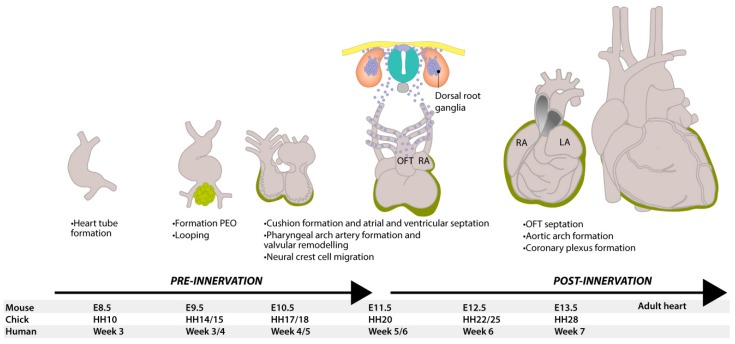
Development of the heart and neural tube in mice. At E8.5, the murine heart is a tube with blood flowing in a peristaltic manner from the caudal venous pole to the cranial arterial (outflow) tract. At E9.5, the heart starts looping and, at the same time, epicardial cells from the proepicardial organ (green) start to migrate and cover the outside of the heart. At E11.5, neural crest-derived cells (blue) delaminate from the neural tube and start migrating ventrally and caudally, contributing to many structures, including dorsal root ganglia. They contribute to valvular remodeling and the septation of the pulmonary and aortic vessels, as well as to delivering neurons to innervate the heart. When the heart has finished looping, the inflow and outflow tract are both found at the base of the heart and the electrical conduction now has an apex-to-base direction. DRG = dorsal root ganglion, HH = Hamburger and Hamilton stage, LA = left atrium, LV = left ventricle, OFT = outflow tract, RA = right atrium, RV = right ventricle, PEO = proepicardial organ.

The adult heart is a four-chambered muscular organ that maintains the pulmonary and systemic circulations. The cardiac muscle is self-exciting and has its own cardiac conduction system, consisting of specialized cardiomyocytes and conduction fibers which initiate rhythmic contractions. Development of the heart is conserved between vertebrate species [9] and, in the sections below, we will largely refer to mouse and chicken embryological stages, extrapolated to the human situation. In Figure 1, relative ages are given for mouse, chick and human development. At mouse embryonic day (E) 8.0, the developing heart can be recognized as a primary heart tube (Figure 1) [10,11]. This hollow tube initially consists of two layers: cardiomyocytes on the outside and endothelial cells on the inside, which are separated by cardiac jelly [12]. At this early time point, the autonomic nervous system has not yet been developed, and blood is pumped through the embryo by peristaltic contraction waves initiated from the caudal (inflow) side of the heart [13]. As development proceeds, the heart tube elongates and undergoes a rightward looping [10,14]. Endocardial cells will undergo endocardial-to-mesenchymal transition (endoMT), and migrate between the two layers to populate the cardiac jelly, resulting in the formation of cardiac cushions at the atrioventricular (AV) canal and outflow tract [15]. Additionally, the cushions will be populated by various cell types, such as epicardium-derived cells [16] and neural crest cells (NCCs) [17], and they will contribute to the atrial and ventricular septation via formation of valves and septa [15].

To generate a third layer of the heart, the heart tube will be covered by an epicardial layer. Epicardial progenitor cells are found at E9.0 (approx. 4 weeks in human, HH14-15 in chick) within the proepicardial organ (PEO), a cauliflower-like cluster of cells at the base of the inflow tract of the looping heart tube (Figure 1) [18,19]. To form the epicardial layer, proepicardial cells migrate towards the heart tube, cover the heart from the inflow tract, attach to the inner curvature and reach the outflow tract at E11.5 [20]. At E12.5, epicardial covering is completed and a subset of epicardial cells lose cell-cell contact, undergo epithelial-to-mesenchymal transition (EMT), resulting in epicardium-derived cells (EPDCs) [21,22]. Lineage-tracing studies in mice have shown that EPDCs migrate into the myocardium and form cardiac fibroblasts and smooth muscle cells [18,23,24,25,26]. Additionally, a role for EPDCs has been suggested in development of Purkinje fibers, which are part of the conduction system [27]. Around E12.5, migrating NCCs will play a role in the development of cardiac innervation and conduction, and contribute to the formation of cushions and septation of the outflow tract (Figure 1) [28]. Together with the atrial and ventricular septation, this results in a four-chambered heart with a complete separation of the pulmonary and systemic circulations.

During cardiac development, the peristaltic movement of the heart tube is replaced by a coordinated contraction, initiated by the sinoatrial (SA) node that passes the electrical activation through the other components of the cardiac conduction system. The initial immature base-to-apex activation pattern will develop into the mature apex-to-base activation pattern that is observed after ventricular septation [29].

The early developing heart is not innervated yet, and the first signs of cardiac innervation during development are found at the dorsal mesocardium at E10.5 in mice [30]. However, in the adult heart, both the SA node and the AV node are extensively innervated, necessary to adjust heart rate to physiological demands.

Although the development of cardiac innervation has been the subject of extensive investigations, the exact processes that govern normal cANS development are not known. Furthermore, the potential contributions of—and interplays between—various cell types are not well understood. In our attempt to link embryonic development to adult pathologies, differences between adult and embryonic cardiac function and anatomy are important. Given the complexity of the cANS, the paragraph below will address the anatomical structures of the cANS in the adult heart.

### 1.2. Anatomy of the Cardiac Nervous System

The nervous system is anatomically divided in two main systems: the central nervous system (CNS) and the peripheral nervous system (PNS). The CNS includes brain and spinal cord, whereas the PNS contains cranial nerves, spinal nerves and ganglia. The PNS is again divided in a somatic (voluntary) and a visceral (vegetative) part, the latter including innervation of the heart. The visceral nervous system contains afferent (sensory) nerves that come from organs and vessels and project towards the CNS. In addition, the visceral system contains efferent (motoric) nerves to the heart, smooth muscle and glands; also known as the autonomic nervous system (ANS).

**Figure 2:**
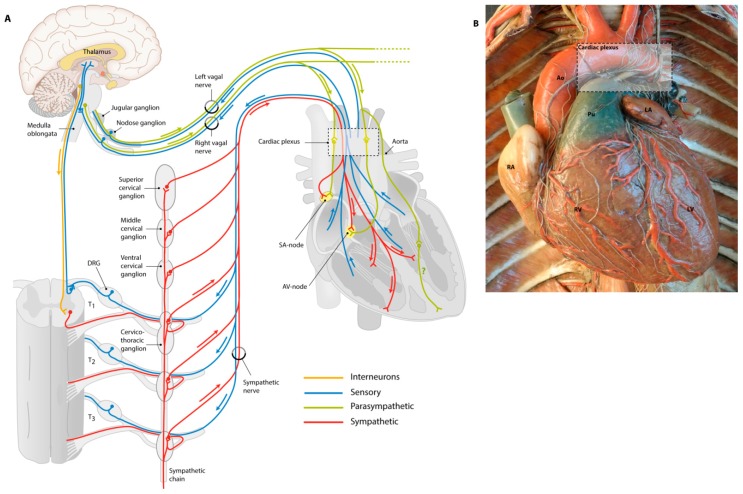
Overview of cardiac innervation. (**A**) Schematic drawing of the cardiac visceral innervation system. Cardiac innervation starts with a signal from the heart or baroreceptors (e.g., on the aorta), relayed by sensory nerves (blue) giving feedback on, for instance, the levels of oxygen, carbon dioxide and blood pressure. The brain will give a signal to parasympathetic or sympathetic nerves to either relax or stimulate the heart. Parasympathetic innervation is achieved mainly via the vagal nerve (green) that will synapse in cardiac ganglia from where postganglionic nerves innervate the SA node and AV node, and potentially ventricular myocytes. Sympathetic neurons (red) start in the grey matter of the spinal cord, where interneurons (orange) from the brain project to the sympathetic neurons. Via the ventral root of the spinal cord, sympathetic nerves synapse in the sympathetic chain, from where postganglionic nerves will enter the heart; (**B**) This wax mold shows how cardiac nerves enter via the cardiac plexus and follow cardiac vessels over the heart. Courtesy of: Museo delle Cere Anatomiche “Luigi Cattaneo”, University Museum System, Alma Mater Studiorum—University of Bologna, picture taken by Dr. E.A.J.F. Lakke. Ao = aorta, DRG = dorsal root ganglion, LV = left ventricle, Pu = pulmonary artery, RA = right atrium, RV = right ventricle.

Adult regulation of cardiac contraction and heart rate is achieved by innervation of the tissues of the cardiac conduction system through the cANS. Anatomically, the cANS is a complex system divided in sympathetic and parasympathetic elements (Figure 2A). All these elements, including the sensory system, are made up of nerve cells (neurons) that contain a neuronal cell body with a nucleus, and extensions (axons and dendrites). When these extensions are arranged and bundled, they form nerves. Nerves of both the sympathetic and parasympathetic cardiac autonomic nerve system originate in the CNS as preganglionic nerves. These nerves synapse within the ganglion and use the neurotransmitter acetylcholine to signal to postganglionic nerves that extend to the target organ. Innervation of target organs is established via postganglionic synapses, where neurotransmitters bind to postsynaptic receptors located on the target organ. Postsynaptic receptors can be either adrenergic receptors (that use catecholamines as ligands) or muscarinic receptors (that use acetylcholine as ligands). The location of ganglions and the main used neurotransmitter is nerve type-specific as will be explained below.

#### 1.2.1. Sensory Nerves Give Feedback

Sensory nerves have a different anatomy and function than the sympathetic and parasympathetic nerves. Their main cardiac function is to give feedback from baroreceptors on the aortic arch to the brain in order for the brain to react and maintain homeostasis. Sensory nerves provide information to the central nervous system and relay information on, for instance, carbon dioxide, blood pressure, and oxygen and sugar levels in the blood. The sensory neuronal body gives rise to axons which are sprouting to both heart and brain. The signal starts in the heart and is directed towards the brain via the neuronal body, in contrast to sympathetic and parasympathetic nerves where the neuronal body also indicates the starting site of the signal. The neuronal body of cardiac sensory nerve fibers lies either within dorsal root ganglia or nodose ganglia, depending on which nerve it accompanies. For instance, the afferent sensory elements that signal to the brain have their neuronal bodies in the nodose ganglion and they accompany the efferent parasympathetic elements of the (mixed) vagal nerve (upper blue lines in Figure 2A). Other sensory nerves follow the sympathetic nerves to the sympathetic chain, from where they split off the sympathetic nerves and move through dorsal root ganglia to the spinal cord (lower blue lines in Figure 2A). Sensory nerves will give their input from the heart to the brain, upon which the brain reacts by modulating the output towards the heart, which is regulated by the parasympathetic and sympathetic nerves.

#### 1.2.2. Sympathetic Nerves Have a Stimulating Effect

Cardiac sympathetic preganglionic nerves arise from the cervical and thoracic portions of the spinal cord from where they project to two chains of sympathetic ganglia parallel to the spinal cord (Figure 2A, red line). In these ganglia, the main neurotransmitter to forward the signal from the central nervous system to postganglionic nerve cells is acetylcholine. From sympathetic ganglia in the sympathetic chain, cardiac postganglionic fibers innervate smooth muscle cells (SMCs) from the (cardiac) circulatory system, as well as elements of the conduction system (e.g., SA node and AV node) and ventricular cardiomyocytes in the heart. While acetylcholine is used as the main neurotransmitter by preganglionic neurons, sympathetic postganglionic neurons predominantly use norepinephrine. Activation of the sympathetic system will generate a fight-or-flight response: the heart rate will be increased by stimulation of the conduction system, and contraction force is increased upon stimulation of the ventricles. In the adult heart, the main receptor encountered is the β1-adrenergic receptor, while in neonatal hearts, α1-, β1- and β2-adrenergic receptors are all expressed, with increasing importance for β1-adrenergic receptors during postnatal development [31]. Adrenergic receptors are G-protein coupled receptors that induce calcium-dependent contractility and output in cardiomyocytes. To prevent calcium overload, the G-protein coupled receptor kinase (GRK) type 2 acts as negative feedback by uncoupling the G-protein from the adrenergic receptors (desensitization) [32,33,34]. The expression of GRK2 is essential for cardiac development, as GRK2^−/−^ mice die before E16.0 due to heart failure. Mutated mouse embryos have decreased heart function and show major cardiovascular abnormalities, such as enlarged lumens of ventricles and atria, and reduced compaction and trabeculation [34].

#### 1.2.3. Parasympathetic Nerves for Relaxation

The heart is parasympathetically innervated by branches of the tenth cranial nerve: the vagal nerve. The vagal nerve sprouts from the medulla oblongata through the superior jugular and inferior nodose ganglia, branching along the way and ultimately synapsing in the cardiac plexus (Figure 2A, green line and Figure 2B). Parasympathetic cardiac ganglia are located near to or within the heart and therefore, parasympathetic postganglionic nerves are short compared to sympathetic postganglionic nerves that sprout from the sympathetic chain. Both pre- and postganglionic nerves use acetylcholine as their main neurotransmitters, acting on cholinergic muscarinic receptors. The vagal nerve transmits parasympathetic activity to lower heart rates by innervating the SA node and AV node. The question remains whether ventricular tissue is also directly innervated by parasympathetic nerves; some studies report the presence of cholinergic nerves in the epicardial and endocardial surface of both ventricles [35,36,37]. In addition, it was found that sprouts from the vagal nerve not only innervate the AV and SA nodes, but also directly influence cardiac ventricular function (reviewed in [38,39]). However, the distribution of parasympathetic nerves in the heart and function of vagal stimulation to ventricular myocytes remains to be investigated.

## 2. Catecholamines in the Pre-Innervation Phase of Cardiac Development

**Figure 3:**
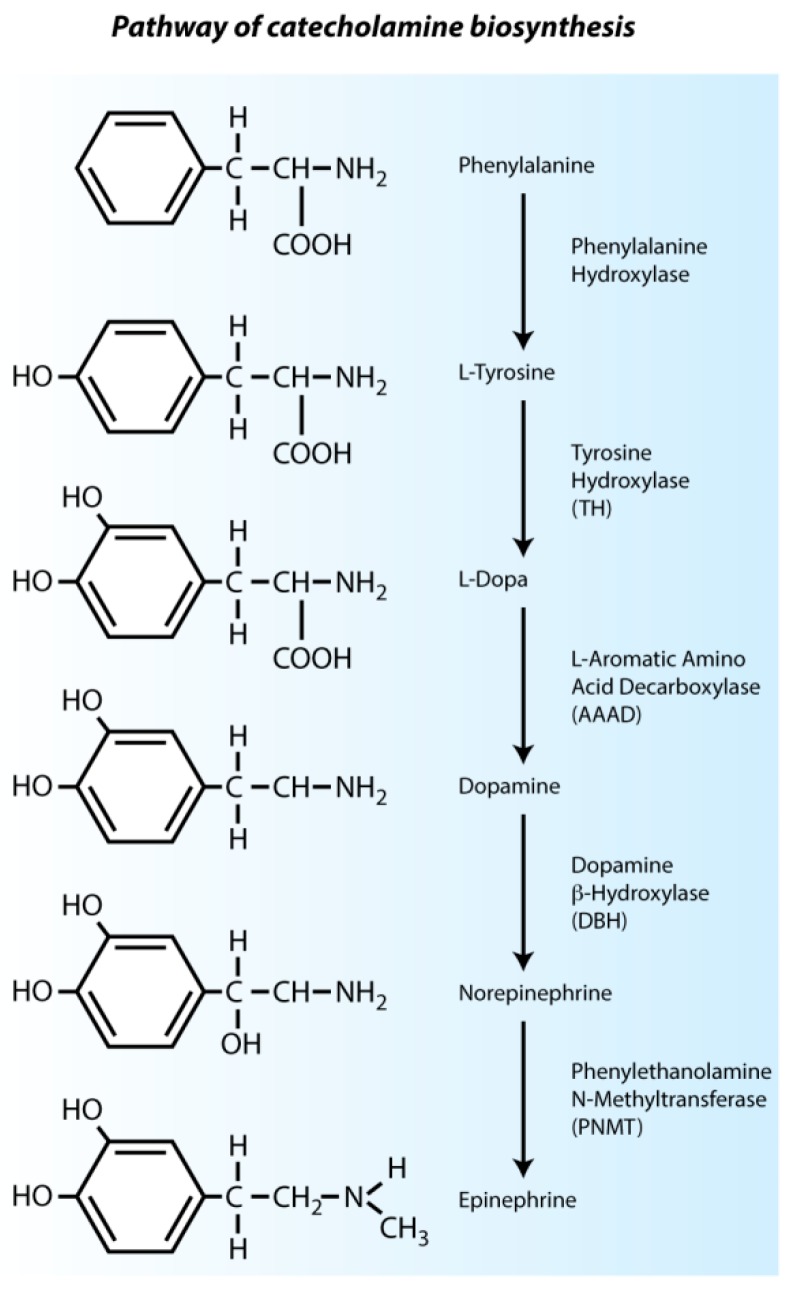
Pathway of catecholamine biosynthesis. Synthesis of epinephrine and norepinephrine is regulated by catecholamine synthesizing enzymes. Synthesis of catecholamines starts with conversion of phenylalanine to l-tyrosine, which is converted to l-dopa by tyrosine hydroxylase (TH). l-dopa is processed to dopamine by l-aromatic amino acid decarboxylase (AAAD), from where norepinephrine is formed by dopamine-β-hydroxylase (DBH). Finally, epinephrine is synthesized by addition of a methyl group to norepinephrine by phenylethanolamine-*N*-methyltransferase (PNMT). Norepinephrine and epinephrine both act as neurotransmitters for sympathetic innervation.

In adult mammals, catecholamines are mainly produced by chromaffin cells in the medulla of the adrenal glands, and to a lesser extent in nerve endings. During embryonic development, catecholamines such as norepinephrine, epinephrine and dopamine are present even before the adrenal glands and cardiac nerves have developed. In this so-called pre-innervation phase, where the (sympathetic) innervation is still lacking, the chicken heart already responds to catecholaminergic stimulation [40,41]. The catecholaminergic biosynthesis pathway is shown in Figure 3. The role of catecholamines in the pre-innervation phase of cardiac development has been extensively investigated and will be discussed below.

### 2.1. Intrinsic Cardiac Adrenergic Cells Are an Early Source of Catecholamines

Early catecholamine expression is essential for (cardiac) embryonic development in pre- and post-innervation phases, and fetal mice have an absolute dependence on norepinephrine from E9.5 to E13.5 [42,43]. When the expression of dopamine-β-hydroxylase *(Dbh)* is disturbed, most homozygous mutant mice die *in utero*, likely due to cardiovascular failure; the surviving mutant mice, moreover, are smaller than their wild-type littermates. Only 5% of the homozygous mice reach adulthood [42]. GATA3 is required for tyrosine hydroxylase (TH) expression and *Gata3* knockout mice die at E11.0 [44]. Disruption of the phenylethanolamine-*N*-methyltransferase *(Pnmt)* gene and the subsequent loss of epinephrine does not affect development of knock-out embryos, likely due to compensation by its precursor norepinephrine [45]. This confirms the dependency of developing embryos on norepinephrine specifically.

The fact that cardiac cells are able to react to early administration of catecholamines indicates that adrenergic receptors are present and fully functional before the development of sympathetic innervation. Endogenous cardiac epinephrine and norepinephrine levels are increased in early chicken development prior to sympathetic innervation [46]. During early cardiac development in both mice and chicken, expression of the catecholamine-synthesizing enzyme PNMT is found throughout the myocardium before its synthesis in the adrenal glands (E15.5) or before development of sympathetic cardiac nerves (E11.5) [46,47,48]. These PNMT-positive cells are now known as intrinsic cardiac adrenergic (ICA) cells and are the potential source of early endogenous catecholamines required for development. ICA cells constitutively release epinephrine, dopamine and norepinephrine in the embryonic mouse heart from E8.5 onwards and synthesize up to one-third of the total cardiac epinephrine levels [45,49,50,51].

**Figure 4:**
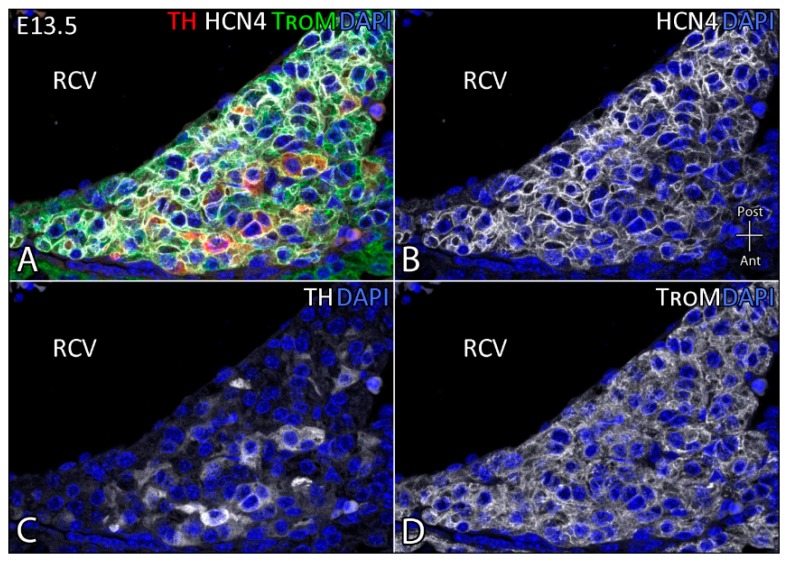
ICA cells within the SA node in the mouse heart at E13.5. The SA nodal region in the mouse heart contains TH-positive cells (**A**); HCN4 expression demarcates the SA node region (**B**); wherein some cells co-express TH (**C**); Tropomyosin (TroM) is used as a general cardiomyocyte marker (**D**). Magn. 63×. RCV = right cardinal vein.

Clusters of ICA cells producing catecholamine-synthesizing enzymes have been reported in regions of the caudal-dorsal atrial region associated with SA node development, and in the AV canal region [47,52]. The intense expression of catecholamine-synthesizing enzymes in the nodal regions is reduced at E16.5 and almost lost at E19.5 in rats. It is more restricted to the upper portion of the ventricular septum, identified as the region where the early His bundle develops at these stages [47]. This suggests that there may be an association between ICA cells and development of the cardiac conduction system. Indeed, derivatives of ICA cells are found to form ventricular myocytes and cardiac conduction cells in the SA node and AV node [45]. Accordingly, we observed expression of the enzyme TH in a subset of cells of the SA node in sequential stages of mouse development, including the pre-innervation stages (unpublished). An example of this TH expression in the developing SA node is given in Figure 4. The SA node is HCN4-positive and, within this region, patches of TH-positive cells are found. These cells are thought to be ICA cells that contribute to the development of the cardiac conduction system.

The origin of ICA cells is still under debate. Morphologically, they do not seem to have a neuronal background and ICA cells are already present at E8.5, before NCCs have invaded the heart [45,47,49]. PNMT^+^ cells appear in the heart at E8.5 in mice, which is a separate cell population from neural crest-derived PNMT^+^ cells that emerge at E10.5 [53]. Furthermore, it seems that the pattern of ICA cells in the heart has no similarity with the distribution of NCCs in the heart and that ICA cells do not migrate from the outside into the heart, but are clustered in the myocardial wall and at junctional regions between atria and ventricles at E9.5 and E11.5 [45,54].

The clinical implication of these cells is indicated both during and after development, in the adult heart. Interestingly, four weeks after sudden denervation due to heart transplantation, the amount of ICA cells, as well as the gene expression of *TH*, *DBH* and *PNMT,* is increased [55]. In addition, ICA cells from the left ventricle express cardioprotective genes such as calcitonin gene-related peptide (*CGRP*) after cardiac transplantation and during ischemia/reperfusion, thereby contributing to damage control [56,57]. This suggests that ICA cells act as a backup system when innervation is lost or under construction, during development or due to pathology.

### 2.2. Response to Epinephrine Administration Is Epicardial Cell Dependent

As mentioned before, chick hearts already respond to catecholamine administration in the pre-innervation phase [40,41]. The presence of epicardial cells seems to be crucial for this early sympathetic response to catecholamines. Administration of epinephrine to the heart gives no electrophysiological response in HH15 chicken embryos, when the heart is still lacking an epicardial layer [41]. When epicardial cells start covering the heart from the PEO at HH19, administration of epinephrine does result in an increased heart rate. Moreover, inhibition of epicardial outgrowth from the PEO in chick will lead to a reduction of response to catecholamine administration at HH24 [41]. The catecholaminergic β_2_-adrenergic receptors that are responsible for this response are found on epicardial cells, in the PEO and at the endocardium at E11.5 in mice (HH21 in chick) [41]. We have found that in mice at E9.5 (equivalent to HH17 in chick), neuron-specific β3-tubulin staining is present in the PEO; the organ from where epicardial cells will cover the heart. At E10.5 (equivalent to HH19 in chick), these β3-tubulin-positive epicardial cells dissociate from the PEO and envelop the myocardium [41]. These data show the importance of epicardial cells in the catecholaminergic response during the pre-innervation phase, although the mechanism is still unclear.

## 3. Neural Crest Cell (NCC) Migration towards the Heart

NCCs are a specific population of multipotent cells that reside at the border regions of the developing neural plate. During the fifth week of human development (HH11 in chick and E8.5 in mice), NCCs undergo EMT and start to migrate from the dorsal neural tube through the embryo, where they contribute to the development of a wide range of cardiac and non-cardiac structures [58]. NCCs from different parts of the neural crest participate in different developmental processes, and a rough distinction can be made between two areas: the trunk and cranial neural crest regions. Some structures are a result of NCC migration from both regions, such as the PNS that contains all nerves and ganglia on the outside of the brain and spinal cord. Sympathetic and parasympathetic nerves (both part of the PNS) are derived from different regions. Trunk NCCs (corresponding to somites 8–28) migrate and form the sympathetic ganglia [59]. Cranial (or vagal) NCCs (corresponding to somites 1–7) participate in the development of ectomesenchymal cells in the head and neck, which contribute to the development of skeletal structures of the face [60]. Furthermore, cranial NCCs provide cardiac NCCs that participate in cardiovascular development [61,62]. Cardiac NCCs are a subpopulation of cranial neural crest, residing between the rhombencephalon (otic placode) and somite 4. They contribute to outflow tract septation and pharyngeal arch artery formation, and supply neuronal and mesenchymal derivatives, SMCs and cardiac parasympathetic ganglia [30,63,64,65]. Using quail chick chimeras, it has been established that cells from the cardiac neural crest also play a critical role in the development of the PNS of the heart [66]. The contribution of NCCs will be discussed further in this review.

### 3.1. NCC Migration to Sympathetic Ganglia Is Regulated by Trophic Factors

**Figure 5:**
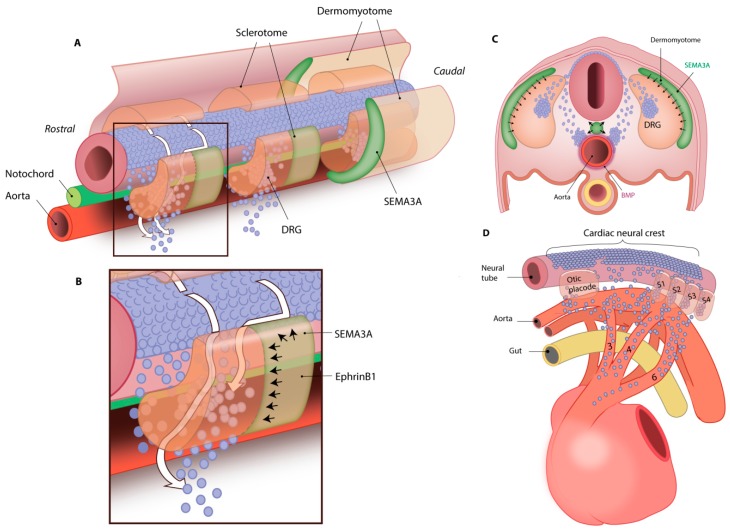
Migration route of the NCCs. (**A**) NCCs (blue) migrate through and ventral to the sclerotome, guided by repulsive neurotrophin SEMA3A from the dermomyotome; (**B**) Inset of A. NCCs are guided by repulsive proteins SEMA3A and EphrinB1 over the rostral side of the sclerotome; (**C**) Within the sclerotome, some NCCs will form dorsal root ganglia (DRG), while others will travel to the aorta. There they will form sympathetic chain ganglia, stimulated by BMPs expressed by the aorta; (**D**) The cardiac NCCs from the cardiac neural crest will follow the vessels into the heart. Cells in the outflow tract will also secrete BMPs to induce differentiation of the NCCs.

NCCs migrate through the ventral side of somites towards the dorsal aorta and pharyngeal arch arteries at E8.5 in mice, and along the way aggregations of NCCs become the sympathetic chain and dorsal root ganglia (Figure 5) [67]. To guide the migrating NCCs over the ventral side of somites towards their destination, attracting and repelling neurotrophic factors regulate neuronal growth (Figure 5A). Several axon guidance proteins are known, such as Semaphorin (SEMA), Netrin and Ephrin. NCCs express the Ephrin receptor EphB2, whereas its ligand EphrinB1 is expressed by the caudal side of somites. EphrinB1 has a repulsive effect on EphB2 receptors, forcing the sympathetic trunk NCCs to move the somites at the rostral side (Figure 5B) [68]. In addition, NCCs express the SEMA receptors Plexin-A1/2, Plexin-D1 and Neuropilin-1/2 (NRP1/2) on their cell surface [69]. Two of their ligands, SEMA6A and SEMA6B, are expressed in the dorsal neural tube and repel NCCs. In contrast, SEMA3C is expressed by myocardial cuff cells in the outflow tract and attracts NCCs from a distance [70,71]. Neurotrophin-3 (NT-3) is one of the more proximal guidance cues secreted by arterial vascular smooth muscle cells (VSMCs). NT3 induces sympathetic axon guidance towards the heart alongside arteries [72]. This indicates that the migration of NCCs contributing to sympathetic innervation is closely orchestrated by several factors. In addition, migrating NCCs communicate directly with neighboring NCCs, regulating the patterning of sympathetic ganglia. *N*-cadherin is an adhesion protein that is expressed at two stages of neural crest cell development: by the neural tube during neural tube formation and by a subpopulation of migrating NCCs [68]. Some migrating NCCs will express *N*-cadherin and adhere to each other, forming aggregations at the site of the developing ganglia. Connexin 43 (Cx43) is a gap junction protein that forms channels between neighboring cells; the rate of NCC migration to the outflow tract is regulated by Cx43 expression. Inhibition of Cx43 function decreases the migration rate and the amount of NCCs that arrive at the outflow tract. When Cx43 expression is enhanced, the amount of NCCs at the outflow tract is increased [73,74]. Motility of the migrating NCCs is also inhibited by SEMA3A that is secreted by the dermomyotome and by the notochord (Figure 5C) [75,76]. This repulsive protein pushes Nrp1-expressing NCCs through and alongside the sclerotome, but also inhibits migration beyond the notochord [76]. This promotes aggregation of the NCCs, thereby inducing the formation of dorsal root ganglia and sympathetic chain ganglia (Figure 5C) [74,76,77]. The importance of trophic factors in NCC migration is shown in *Nrp1* and *Sema3a* null mice, where defective trunk NCC migration results in abnormal development of sympathetic ganglia and the development of sinus bradycardia [77,78].

### 3.2. NCC Migration to Future Parasympathetic Ganglia Locations

Cardiac NCCs that migrate from the cranial (also called vagal) neural crest are responsible for the formation of cardiac parasympathetic ganglia [63,64,79]. Parasympathetic NCCs do not form a paravertebral ganglionic chain as is the case with sympathetic innervation, but migrate through and lateral to the somites directly towards their target organ. They form parasympathetic ganglia after reaching the heart around E12.5 [30]. Interestingly however, ablation of the neural crest does not affect cholinergic innervation, suggesting that the parasympathetic system may have an alternative non-neural crest source for cholinergic neurons [80]. In line with this finding, lineage tracing of Wnt1^+^ neural crest cells in mice showed that neurotrophin receptor (NTR) p75-positive cells are already present in the dorsal mesocardium at E10.5, two days before the NCCs have reached that area. This alternative source of cells is thought to be from the nodose placode in the third pharyngeal arch, since parasympathetic ganglia are found to obtain cells and form cholinergic neurons derived from this area when the cardiac neural crest is ablated [30,80].

## 4. Differentiation of Neural Crest-Derived Cells

**Figure 6:**
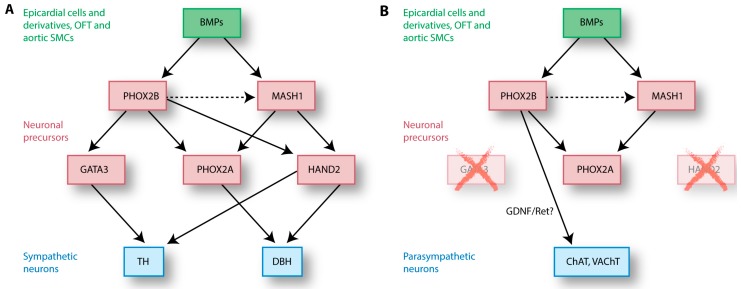
Differentiation of neural crest cell-derived neuronal precursors is induced by intrinsic and extrinsic factors. (**A**) Bone morphogenetic proteins (BMPs) secreted by epicardial cells and vascular cells induce differentiation of NCCs to sympathetic neurons by initiating the expression of paired-like homeobox (PHOX)-2B and mammalian achaete-scute homolog (MASH)-1 in the NCCs. PHOX2B induces the expression of tyrosine hydroxylase (TH) via GATA3, and has a positive effect on MASH1 expression. MASH1 and PHOX2B both induce the expression of dopamine-β-hydroxylase (DBH) through PHOX2A and heart and neural crest derivatives expressed 2 (HAND2), the latter being essential for sympathetic differentiation; (**B**) The expression of Gata3 and HAND2 is lost in parasympathetic precursors, as is the downstream expression of TH and DBH. The expression of parasympathetic markers ChAT and VAChT is probably regulated via glial cell line-derived neurotrophic factor (GDNF)/Ret. OFT = outflow tract, SMC = smooth muscle cell.

After NCCs have delaminated from the neural tube and reached the dorsal aorta at E9.5 and the outflow tract at E10.5 (Figure 5D), the next step is to differentiate towards functional (cardiac) neurons [30,64,81,82]. This differentiation coincides with migration of the NCCs, as NCCs will pick up locally secreted factors that induce differentiation during their journey (Figure 5C) [83]. As a result, NCCs become more dedicated to one specific cell type and lose a part of their multipotency as they migrate through the body to their final destination. The moment NCCs reach the heart, a subpopulation is already designated as neuronal precursors and expresses certain neuronal proteins as will be discussed below and in Figure 6.

### 4.1. Do Sympathetic and Parasympathetic Neurons Share a Common Precursor?

NCCs have been isolated from rat neural tubes at E10.5. In vitro, these cells can differentiate into neurons, Schwann cells and myofibroblasts upon stimulation with growth factors such as neuregulin and bone morphogenetic protein (BMP)-2/4/7 [84,85,86]. The dorsal aorta expresses all of these three BMPs around the time NCCs arrive at this site, making the dorsal aorta a likely source of BMPs that initiate NCC differentiation (Figure 5C) [85,86]. Interestingly, BMP2 expression in the heart is found in epicardial cells and EPDCs, coronary vascular cells, the outflow tract and in SMCs of the aorta [87], suggesting that the epicardium and its derivatives play a crucial role in the development of cardiac innervation. BMP release initiates a transcriptional cascade in NCC-derived neuronal precursors by activation of maturating genes, including the basic helix-loop-helix (bHLH) transcription factor mammalian achaete-scute homolog (MASH)-1 (Figure 6) [85,88]. MASH1 expression is restricted to neuronal precursors, and aggregations of MASH1-positive cells are observed in sympathetic ganglia around the aorta and in parasympathetic cardiac ganglia [89,90,91,92]. MASH1 is vital for autonomic neurogenesis and maturation of both sympathetic and parasympathetic peripheral autonomic neurons [89]. It is likely that cholinergic and noradrenergic neurons share a common precursor, since sympathetic neurons can change their fate to a cholinergic phenotype when stimulated with BMP2 in vitro [93]. It appears that local BMP2 levels determine the outcome of differentiation: NCCs respond to low BMP2 levels by differentiating into parasympathetic neurons, whereas high levels of BMP2 induce differentiation towards sympathetic neurons [93]. In contrast with this finding, an overexpression of BMP does not lead to an increase of TH/DBH-expressing cells compared to the proportion of cholinergic cells [94]. Interestingly, a transient expression of noradrenergic genes is found in cells before they acquire a cholinergic phenotype, and some cells express both sympathetic *TH* and cholinergic vesicular acetylcholine transporter (*VAChT)* at HH24 [94]. These results suggest that NCCs differentiate towards a common neuronal precursor, before acquiring their final neuronal phenotype. NCC differentiation towards sympathetic and parasympathetic neurons is dependent on the expression of their neurotransmitters and synthesis enzymes, as will be discussed in the paragraphs below.

### 4.2. NCC Differentiation into Sympathetic Neurons

Sympathetic neurons distinguish themselves from parasympathetic neurons by the expression of norepinephrine-synthesizing enzymes, such as TH and DBH (Figure 6). These enzymes are produced by the neuronal precursors (NCCs) themselves during differentiation towards neurons, induced by factors secreted by surrounding cells. Several factors are involved in this narrowly orchestrated process (Figure 6A). MASH1 induces expression of transcription factor Paired-Like Homeobox (PHOX)-2A, which together with BMP-induced PHOX2B enhances expression of the enzyme DBH [95,96]. PHOX2B expression is independent of MASH1, whereas maintenance of MASH1 expression is dependent on PHOX2B [97]. Mutation of *Phox2b* in mice disrupts expression of MASH1, but also expression of the glial cell line-derived neurotrophic factor (GDNF) receptor Ret, which is required for parasympathetic ganglion development [97,98]. A mutation of *Phox2b* therefore affects the development of the entire autonomic nervous system. Downstream of PHOX2B, GATA3 is a sympathetic neuron-specific transcription factor and has no (known) effect on parasympathetic development. This transcription factor is important for TH expression, and TH itself is essential for development of the sympathetic nervous system. When *Gata3* is knocked down, mutant mouse embryos have reduced *Th* and *Dbh* mRNA expression and die at E11.0 due to norepinephrine deficiency [44]. The bHLH transcription factor HAND2 (heart and neural crest derivatives expressed 2) is specifically required for the formation of a noradrenergic phenotype of the sympathetic ganglia by induction of TH and DBH [99,100]. After neuronal formation, HAND2 also plays a role in maintenance of the noradrenergic TH- and DBH-expressing properties of the cANS in adult mice [94,101]. Interestingly, when *Hand2* is knocked-out, the expression of cholinergic markers VAChT and choline acetyltransferase (ChAT) is increased in sympathetic neurons, suggesting that a switch from noradrenergic to cholinergic phenotype is possible [101]. Again, this finding strengthens the idea of a common neuronal precursor of the sympathetic and parasympathetic neurons.

### 4.3. NCC Differentiation into Parasympathetic Neurons

Differentiation of parasympathetic neurons is similar to sympathetic neuron maturation, with the main difference found in the expression of norepinephrine-synthesizing enzymes (Figure 6B). The proteins BMP, MASH1 and PHOX2B are important for the differentiation of NCCs towards both sympathetic and parasympathetic neurons. Indeed, at early stages, the differentiation of neuronal precursors towards cholinergic parasympathetic nerves does involve DBH and TH expression. However, parasympathetic neuron precursors do not express *Gata3* and *Hand2* (Figure 6B) [94,100]. The downregulation of *Gata3* and *Hand2* is probably the cause of the rapid loss of DBH and TH at later stages, which induces differentiation toward parasympathetic neurons [94]. The cause of the absence of HAND2 and GATA3 in parasympathetic precursors remains to be elucidated.

Development of parasympathetic cardiac ganglia is not disturbed in *Phox2a*-deficient mice [102]. In contrast, the formation of parasympathetic ganglia is affected in *Phox2b* mutant mice and no parasympathetic ganglia are found at all at E13.5 [97]. As mentioned before, PHOX2 proteins are important for Ret expression, and the expression of GDNF and its receptor are required for parasympathetic ganglion development [98]. How this process may be regulated exactly is still unknown. The parasympathetic pathway is less understood than the sympathetic differentiation pathway, and the manner in which factors interact needs to be investigated further to understand the complete process of parasympathetic differentiation.

### 4.4. Small Intensely Fluorescent (SIF) Cells Act as Interneurons in Autonomic Ganglia

Autonomic ganglia are not only comprised of nerve endings and neuron cell bodies, but also contain satellite glial cells (that cover neuronal cell bodies and supply nutrients to neurons) and small intensely fluorescent (SIF) cells. SIF cells are neural crest-derived granulated cells that reside in autonomic ganglia and are thought to act as interneurons between principal pre- and postganglionic nerves [103,104,105]. Their name is explained by the fact that they are small cells (10–20 µM diameter) that stain highly positive for catecholamine-synthesizing enzymes such as TH, DBH and PNMT [106]. SIF cells are innervated by pre-ganglionic nerve endings upon which they release catecholamines from their granules and modulate ganglionic synapsing [107,108]. In rats, these SIF cells are present from E13.0 in sympathetic ganglia, then levels decline after birth, to increase again from P14-21 to adult levels [109]. This suggests that together with ICA cells, they also deliver catecholamines to the heart prior to the development of the adrenal glands. In the heart, SIF cells are mainly found within the cardiac ganglia in the subepicardial layer, but are also found in the epicardial layer [110]. A distinction is made between two types of SIF cells, based on their phenotype and distribution [107]. In vivo, type I SIF cells act as interneurons and are found to be more dispersed and solitary, with long axons parallel to ganglionic neurons. Type II SIF cells form dense clusters near blood vessels and are thought to act as endocrine (chromaffin) cells, releasing their granule content directly into the blood circulation upon stimulation [107,111]. In vitro, SIF cells take on both type I and type II phenotypes, depending on culture conditions. SIF cells display a type II-like (endocrine) phenotype when ganglia are cultured with low hormone and non-neural cell factors [112]. When sympathetic ganglia are stimulated with corticotropic hormones, the induction of type I SIF cell number in these ganglia is identified by an increased secretion of epinephrine, TH and DBH [112,113,114]. When this glucocorticoid stimulation is supplemented with nerve growth factor (NGF) treatment on sympathetic ganglia, the expression of TH and DBH by the type I-like SIF cells is even further increased and the SIF cells show a more neuron-like phenotype [112,114]. These findings suggest that glucocorticoids increase the number of type I SIF cells and facilitate the differentiation of SIF cells to neurons through NGF, which leads to an increase of catecholamine levels in autonomic ganglia.

Research in frogs showed that the development of cardiac parasympathetic ganglia is correlated with the formation of SIF cells, and SIF cells are derived from NCCs in similar neural crest regions [115]. The presence of catecholamine-positive SIF cells in the cardiac ganglia may explain why parasympathetic ganglia stain positive for both cholinergic and adrenergic markers [116,117]. Indeed, only the small cells are positive for catecholamine staining in parasympathetic cardiac ganglia, which may be SIF cells [118]. Since SIF cells project to principal neurons, it is possible that they modulate activity of the SA node and AV node indirectly. More research is needed to elucidate the function of these catecholamine-secreting SIF cells and their role in cardiac autonomic development.

## 5. Neuronal Survival and Patterning

Once NCCs have reached their destination and have differentiated into neurons, survival factors are secreted by cells of the target organ such as cardiomyocytes, VSMCs or satellite glial cells. These factors are secreted to ensure developed neurons are matched to their target: cells that do not receive enough of survival factors will undergo apoptosis. Below, three of the major factors and their associated cells are described.

### 5.1. Nerve Growth Factor Is Important for Sympathetic Neuron Survival

NGF is a neurotrophic guidance cue, but also acts as a maintenance survival factor in embryonic and adult life. It is mostly present in a proNGF isoform in sympathetic neurons and becomes active NGF after extracellular cleaving by neuron-secreted matrix metalloproteinases (MMPs) [119,120]. When sympathetic nerves have reached their destination (or target site) in the heart, NGF secreted by cells in the target region plays an important role in survival of the neurons. The ratio of proNGF versus mature NGF determines axonal degradation or survival. ProNGF has a higher affinity to neurotrophin receptor p75 of the apoptosis pathway, meaning high local expression of pro-NGF will lead to apoptosis of axons in that area. In contrast, NGF has a higher affinity to receptor TrkA, thereby inducing survival of the axons in that particular area [119,121,122].

Expression of *Ngf* is first detectable in mouse embryos at E12.0 in the cervical ganglion and the expression increases until E14.0. A drop in expression levels around birth is followed by a second peak at P8, after which the expression decreases and reaches its mature levels around P21 [123]. In the heart, expression levels of *Ngf* are likely related to the extent of innervation: *Ngf* expression is much higher in the more densely innervated atrium than in the ventricle [124]. Cardiomyocytes are well-innervated and are, together with the earlier mentioned VSMCs, a major source of cardiac NGF. Cardiomyocyte-derived NGF expression is promoted by VSMC-produced endothelin-1, making endothelin-1 an important regulator of cardiac sympathetic innervation [1,125]. Additionally, sympathetic and parasympathetic neurons are able to synthesize NGF themselves and retrogradely transport NGF from its target to the neuronal cell body. The function of this neuron-derived NGF expression is however still under debate [123,126,127,128,129]. Parasympathetic cardiac ganglion neurons can also release NGF, and this release is induced through β-adrenergic activation, regulated by sympathetic innervation [129].

Interestingly, sympathetic and parasympathetic neurons in ganglia are lost in mice with disrupted *Ngf* gene or protein expression, but even without NGF the neurons survive until at least E16.5 [72,130,131,132]. In addition, more than half of the dividing neurons die in serum-free cultures and cannot be rescued by NGF addition [133], suggesting that survival of the other half of the neurons is not NGF-dependent but depends on another factor present in serum. These data suggest that although NGF is an important survival factor, other factors must also play a role in sympathetic neuron survival both in vitro and in vivo.

### 5.2. Neurotrophin-3 is an Alternative Sympathetic Survival Factor

One of the alternative candidates for sympathetic neuronal survival is NT-3, which roughly follows the same expression pattern as NGF during development [72]. NT-3 is highly expressed by cells of blood vessels adjacent to sympathetic ganglia, stimulating axon guidance [72]. The main difference between NGF and NT-3 is their tyrosine kinase (Trk) receptor and its expression. Rat embryonic sympathetic neuroblasts (dividing neuronal precursors) do not carry the NGF receptor *Trka*, but they do express the NT-3 receptor *Trkc*. Furthermore, embryonic neuroblasts do not respond to NGF, while neonatal rat neurons are dependent on NGF in vitro and do not respond to NT-3 [133]. This suggests the presence of a switch in survival dependency from NT-3 to NGF during development, related to a switch in receptor expression. This shift becomes apparent from E17.5 onwards, when less TrkC but more TrkA is expressed on the neuronal cell surfaces. When comparing cell recovery rates of these neurons at different time points in vitro, less neuroblasts respond (and thus survive) upon NT-3 administration and more respond to NGF over time [133].

Although these results suggest that NT-3 is required for neuroblast survival in vitro, Francis et al. found contrasting in vivo data. In developing mice, NT-3 is only necessary for postmitotic neuron survival, as is the case with NGF [72]. No difference in neuroblast counts between E15.5 wild-type mice and NT-3-deficient mice was found, while at P0 a significant decrease of neurons in NT-3-deficient mice was detected [72]. Therefore, although contrasting to the in vitro data, it seems that NT-3 is not required for neuroblast survival, but only for mature neuron survival in vivo.

The contrasting outcomes about the role of NT-3 in neuroblast survival could be due to several factors. The divergence may be explained by (1) the difference between animal models, although mouse and rat development are very similar; (2) the possibility that NT-3 does indeed influence survival of neuroblasts, but only at a later stage than E15.5 and before the dependency switch at E17.5; (3) the idea that in vivo, loss of NT-3 is compensated by other neurotrophic factors and that NT-3 has a simultaneous or sequential interplay with NGF; and (4) the involvement of receptors other than TrkA and TrkC. For instance, when *Trkc* is knocked-out, more neurons survive compared to mice without *Nt-3*, suggesting NT-3 may signal through alternative receptors as well [134]. More research is necessary to address these uncertainties.

### 5.3. Parasympathetic Neuronal Survival by Glial Cell-Derived Neurotrophic Factor and Family Members

Parasympathetic neuron survival is less well understood than sympathetic neuron survival. The role of the four glial cell-line-derived neurotrophic factor (GDNF) family of ligands (GFL) and their receptors are only some of the few factors associated with parasympathetic neuronal development and survival. Although the name suggests that glia cells in ganglia are solely responsible for the expression of GDNF and family, these factors are also expressed by cardiomyocytes [135]. Different GDNF-family ligands bind to different α-GDNF family receptors (GFRα): GDNF binds to GFRα1, Neurturin (NTN) binds to GFRα2, Artemin (ARTN) to GFRα3 and Persephin (PSPN) to GFRα4 [136]. GFRα receptors act as co-receptors to the tyrosine kinase receptor Ret and mediate binding of the GFL to Ret on the same cell surface [137]. Activation of the Ret kinase inhibits apoptosis and promotes survival through Ret-induced AKT signaling, making apoptosis and survival of neurons dependent on the presence of GFRα receptors [138].

In cardiac ganglia, the temporal expression pattern differs amongst cell types. *Ret* and *Gfrα2* are highly expressed in the neurons of cardiac ganglia at E18.0 and P21. In contrast, the non-neuronal glia cells in the cardiac ganglia express *Gfrα1* and *Gfrα3* at these time points [139]. *Gfrα1* expression is mainly found in the semilunar valves, and in the walls of the aorta and pulmonary trunk at E18.0 and P21, and low expression is found in the myocardium at E18.0 but not at P21 [139]. The role of GDNF and GFRα1 in development is demonstrated by the fact that deficient mice die at birth due to the lack of kidneys and disturbed development of innervation [140,141,142]. When *Ntn* or its receptor *Gfrα2* are knocked-out in mice, the animals survive, but develop aberrant parasympathetic innervation [143,144]. Both *Ret* and *Gfrα2* deficiency in mice leads to disturbed development of cardiac parasympathetic innervation as is shown by a decreased number of cardiac ganglia and a lack of cholinergic innervation to the AV node and ventricular conduction system [139]. Since GFRα2 is the receptor to NTN, it is expected that NTN acts as a survival factor for parasympathetic neurons, as is indeed the case in all kinds of organs, such as cranial and ciliary parasympathetic ganglia in the head region. However, the role of NTN on parasympathetic neuron survival in the heart has not been elucidated to our knowledge [98,143,145,146].

## 6. Parasympathetic Cardiac Innervation Precedes Sympathetic Cardiac Innervation

Even though the migration and differentiation of NCCs has been extensively described, pinpointing the first innervation of the heart has proven to be difficult. As mentioned in Section 4, sympathetic and parasympathetic neurons may differentiate from a common precursor neural crest-derived cell population. A difference between early sympathetic and parasympathetic precursors in the heart has not been made and both cell types follow the same route into the heart, making distinction between the two cell types problematic. In addition, species-specific differences make it difficult to determine the exact timing of the developmental stages of cardiac innervation. Nevertheless, the order of innervation events is clear. It has been shown that parasympathetic vagal innervation precedes sympathetic and sensory innervation [64,147,148]. Therefore, below we will first discuss the development of parasympathetic innervation.

### 6.1. The Parasympathetic Pre-Ganglionic Nerve System Is Established by the Vagal Nerve

As mentioned before, NCCs arrive at the OFT around E10.0–10.5 at the arterial pole. These cells have migrated from the cardiac (or so-called vagal) neural crest, differentiating to vagal nerve branches during their migration towards the heart. These nerve branches are used by other NCCs to reach and enter the heart via the dorsal mesocardium around the pharyngeal arch arteries (Figure 5D) [30,64]. The vagal nerve gives off several branches to the heart: branches at the venous side of the heart that reach the sinus venosus, and branches that will arrive at the arterial pole around the same time. Staining for the early neuronal differentiation marker NF160D showed NCC-derived vagal neurofilaments present at the venous pole of the heart of E12.5 mouse embryos [30]. These vagal (preganglionic) nerve fibers and cardiac ganglia are identified as cholinergic neurons by staining positive for VAChT, with an abundance of parasympathetic neurons in the cardiac plexus [30]. 

### 6.2. Parasympathetic Ganglia Are Located Near the Heart and Connect to Short Postganglionic Nerves

The human adult heart contains around 700–900 cardiac parasympathetic ganglia, clustered together in subplexuses which are mostly found in the subepicardium of the atria [149]. In the chick, parasympathetic ganglia are morphologically distinguishable from nerve fibers from stage HH35 [64]. Postganglionic nerves sprout from cardiac ganglia, and extend to the SA node and AV node to innervate the conduction system (Figure 2A, green lines). In addition, parasympathetic nerve fibers in the atrioventricular sulcus penetrate the myocardium at the dorsal and ventral side of the interatrial septum [64].

### 6.3. Developing Sympathetic Innervation Follows Parasympathetic Tracts

Subsequent sympathetic cardiac innervation is established in chick-quail chimeras from HH37–HH40, when TH-positive postganglionic fibers move alongside the previously developed vagal tracts to enter the heart through the cardiac plexus at the base of the heart (Figure 2) [149,150]. These fibers are connected to three main levels of the cervical sympathetic trunk: superior, middle and lower cervical sympathetic ganglia [150]. TH-positive nerve fibers are found at HH42 in cardiac ganglia and near the AV node region, as well as in the ventricular myocardium (Figure 2A, blue lines) [48,150].

## 7. Sympathetic Cardiac Axons Follow Coronary Vessels through the Heart

After entering the heart from the cardiac neural crest via the vagal tracts (Figure 5D), sympathetic cardiac nerves are found to sprout parallel to coronary vessels (Figure 2B), and cells contributing to the development of coronary vessels indirectly regulate development of cardiac innervation [151]. During development, coronary vessels attract cells that secrete factors to guide axons through the subepicardium and to induce invasion into the myocardium.

### 7.1. Sympathetic Axon Guidance over Coronary Vessels

**Figure 7:**
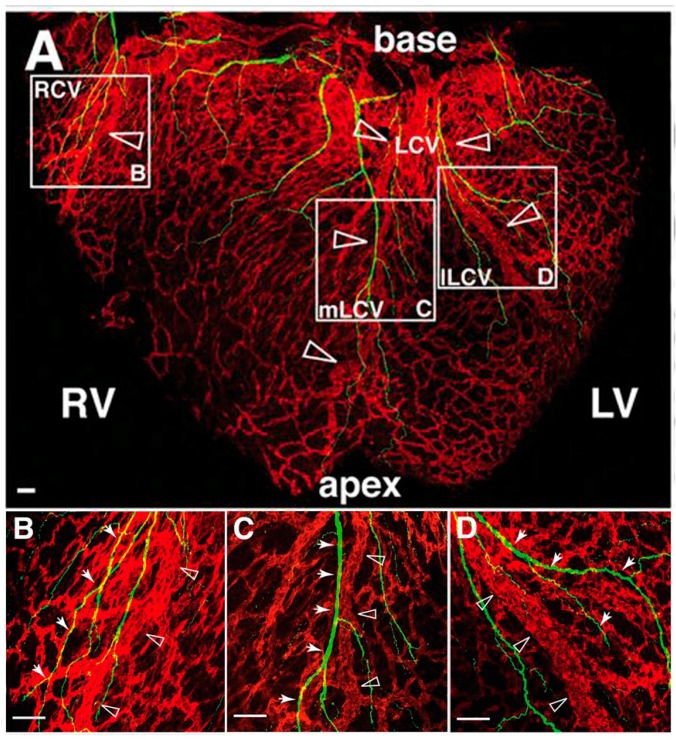
Sprouting axons follow the developed coronary veins. Dorsal views of whole-mount hearts stained for neuronal marker Tuj1 (green) and pan-endothelial marker PECAM1 (red). At E15.5 in mice, Tuj1-positive sprouting axons (green, closed arrow heads) are found in relative close proximity to the larger coronary veins (Red, open arrow heads). RCV = right cardiac vein, mLCV = medial branch of the left cardiac vein, lLCV = lateral branch of left cardiac vein, LV = left ventricle, RV = right ventricle. Adjusted from [151], with permission from Nam, et al., *Development*; published by The Company of Biologists Ltd., 2013.

Although the cellular contribution to the developing coronary vessels is under debate, the link between coronary vessel development and nerve axon growth over the heart is well established. After entering the heart at the cardiac plexus, neurons extend to the apex parallel to the large coronary veins using the subepicardium (Figure 7) [151]. Again, neurotrophic factors are important as a balance between attracting and repulsive guidance cues has to be established to push axons forward over the vasculature. During vasculogenesis, large developing EphB4^+^ veins recruit VSMCs originating from epicardial cells and/or NCCs [24,25,152]. These VSMCs secrete neurotrophic factors to guide extending axons alongside the surface of the cardiac veins [151]. One of these factors is NGF, the primary attractant required for sympathetic axon invasion in the subepicardium of the developing heart [151]. At the same time, from E12.0 to E15.0, the neurorepulsive factor SEMA3A is highly expressed in the subendocardium, thereby generating a balance of attractive and repulsive cues necessary for establishing the epicardial-to-endocardial gradient of sympathetic nerves [77]. At E15.5, the sympathetic nerves moving along the coronary veins have reached the apex at the dorsal side of the heart. At the same time, the NGF-expressing VSMCs present on these veins migrate to the deeper myocardial coronary arteries. Subsequently, axons are attracted to the migrated arterial VSMCs and penetrate the myocardium to reach and extend over the coronary arteries [64,151]. By E17.5, NGF expression is lost in venous VSMCs, but NGF expression is persistent in arterial VSMCs in the myocardial tissue [151]. In conclusion, sympathetic nerve development is preceded by coronary vascular development and follows guidance cues secreted by VSMCs.

### 7.2. Parasympathetic and Sensory Axon Guidance

Parasympathetic and sensory axon guidance are thought to follow slightly other paths than the sympathetic axon growth. There is evidence that sensory nerves do not follow vascular paths, but rather that arteries follow the path laid down by sensory nerves [153]. Although these studies have only been done in the mouse limb skin, it does provide an interesting alternative for development of cardiac innervation.

Parasympathetic axon guidance is probably induced by GDNF and its family members, but their role as guidance cues is unclear [75,154]. In in vitro lung explant cultures, it has been shown that the GDNF family influences the direction of axon growth [155]. In addition, the GDNF family plays a role in guidance of vagal neural crest cells along the gut [156]. Unfortunately, parasympathetic axon guidance specifically in the heart remains an enigma, just as this review raises questions about many other currently unresolved topics.

## 8. Conclusions and Clinical Implications

**Figure 8:**
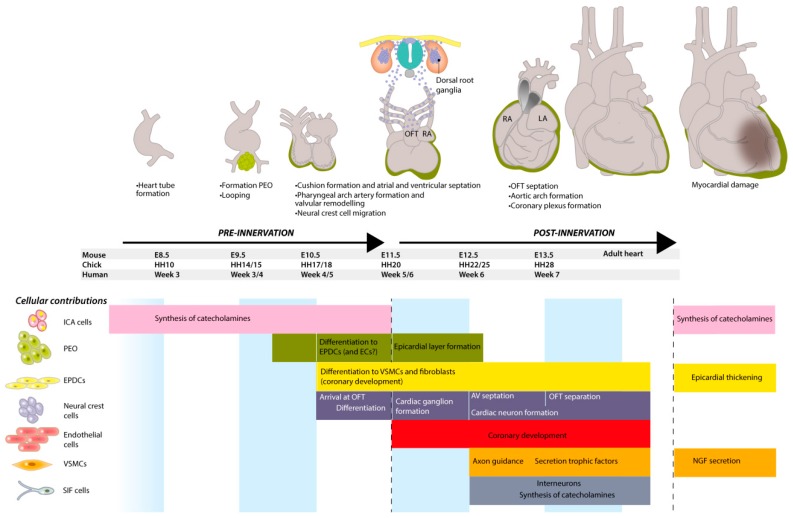
A summary of the development of cardiac innervation and cellular contributions. At every developmental stage, different cell populations are involved. Before cardiac innervation is established, intrinsic cardiac adrenergic (ICA) cells already contribute to the autonomic regulation by synthesizing catecholamines. Additionally, after transplantation, these cells become active again and produce tyrosine hydroxylase (TH) and dopamine-β-hydroxylase (DBH). The proepicardial organ (PEO) is important for the coronary development, but also the source of epicardial-derived cells (EPDCs) and maybe endothelial cells (ECs). The formation of the epicardial layer is a hallmark for cardiac innervation. EPDCs are a major source of several cells, including vascular smooth muscle cells (VSMCs) and fibroblasts, thereby also contributing to the coronary development. Neural crest cells play a multi-facetted role in the development of the heart and cardiac innervation. VSMCs are mainly important for axon guidance through secretion of neurotrophic factors, but are also a player in pathological hyperinnervation by secretion of nerve growth factor (NGF) after myocardial damage. Small intensely fluorescent (SIF) cells can act as interneurons and are together with ICA cells a cardiac source for catecholamines. DRG = dorsal root ganglion, OFT = outflow tract.

Development of the autonomic nervous system (cANS) is a complex process involving many cell types, and is mainly determined by neural crest cell (NCC) delamination, migration and differentiation towards cardiac neurons. Other cell populations have a more supporting role by producing trophic factors to guide cells and axons (vascular smooth muscle cells (VSMCs) and endothelial cells), but also by secreting factors to induce NCC differentiation (VSMCs, epicardium-derived cell (EPDCs)) and survival (VSMCs, cardiomyocytes and neurons). Further support may also come from delivery of cell-derivatives (proepicardial organ (PEO), sinus venosus and EPDCs) that help build guiding vessels (endothelial cells, smooth muscle cells (SMCs) and fibroblasts), or that maintain cardiac function before and during development (intrinsic cardiac adrenergic (ICA) and small intensely fluorescent (SIF) cells). The contributions of the various cell types and their temporal distribution are summarized in Figure 8. All these cells play a temporal and spatial role in the development and maintenance of healthy cardiac innervation. Disturbances in these developmental processes and the factors that are involved may either underlie or be the result of pathological processes that lead to altered innervation. As stated in the introduction, cardiac autonomic dysfunction is a major cause of morbidity and determines prognosis in patients with various cardiac diseases. After an MI (myocardial infarction), several genes and factors are (re)activated that affect myocardial damage, such as epicardium-derived follistatin-like-1 and mineralocorticoid receptor expressed by VSMCs (Figure 8) [157,158].

Secreted factors control the patterning of nerves and in this review, several examples are given of factors involved in this process. For instance, SEMA3A is a neuronal chemorepellent and once affected, mice show disturbed innervation patterning with an imbalance in the epicardial-to-endocardial gradient. Interestingly, these mice exhibit sinus bradycardias and are highly susceptible to induced ventricular tachyarrhythmias [77], suggesting that altered autonomic patterning affects heart failure progression. In addition, neurotrophic and survival factor nerve growth factor (NGF) is a major player in innervation, and the NGF levels are increased in certain pathological conditions. Inflammatory cells such as macrophages, and myofibroblasts synthesize and secrete NGF after an MI [1,159].

Whether or not this increase of NGF is beneficial remains unclear. Some studies show that overexpression of NGF leads to hypertrophic sympathetic ganglia, hyperinnervation and cardiac dilation [1,160]. Ventricular hyperinnervation after MI has been related to sudden cardiac death [1]. In concordance, chemical depletion of macrophages after an MI reduces the levels of NGF in the border zone and inhibits hyperinnervation in rats [161]. Others state that NGF release promotes cardiac repair after MI, by increasing angiogenesis and cardiomyocyte and endothelial cell survival [162]. In either case, the major role of a functional cardiac autonomic innervation system in healthy and diseased situations is emphasized. The importance of the cANS in pathological conditions defines the urge to recover healthy innervation after cardiac injury. This matter has been addressed by White et al. using a model of apical resection in neonatal mice. In contrast to adults, neonatal mice are able to regenerate cardiac tissue after injury, and it seems that reinnervation of the damaged tissue is likely to be an essential step in this process [163].

During chronic heart failure, the sympathetic nervous system is hyperactivated and the increased catecholamine levels ultimately result in detrimental effects on cardiomyocytes. This hyperactivation of the sympathetic nervous system also leads to increased levels of GRK2 and GRK5, related to desensitization of the adrenergic receptors [164,165]. This makes both β-adrenergic receptors as well as GRK2/5 therapeutical targets during chronic heart failure [166,167,168].

The role of the sympathetic nerve system in pathology became even more elusive when researchers found that uninjured rats develop cardiac fibrosis and myocardial injury upon 6-hydroxydopamine-induced sympathetic denervation [169]. When a neuroprotective compound used to treat neuropathies is added during the sympathectomy, the rats do not develop cardiac injury, showing that autonomic denervation is indeed the cause of the injury [169]. Clinically, these observations are very promising as it implies that it is feasible to search for compounds that are able to regulate cardiac sympathetic innervation and denervation.

Even though the cardiac development of several vertebrates is comparable, the postnatal change in heart rate during development may differ between mammalian species [170]. The heart rate of large animals, including humans and rabbits, decreases during development [171,172,173,174], whereas the heart rate of mice and rats increases postnatally [170,175,176]. Heart rate can be induced either by increasing sympathetic regulation or decreasing parasympathetic regulation. It seems that the heart rate of newborn mice is mostly regulated by sympathetic activity [175], whereas regulation of the heart rate in large mammals, including humans, is sympathetic before birth, but dominance shifts to parasympathetic as the vagal tone develops postnatally [177]. These differences in maturation of cardiac innervation and conduction should be taken into account when extrapolating small animal studies to large animals and humans.

In summary, much is still unclear regarding the development of cardiac innervation and the pathologies that are related to it. Further research is warranted to elucidate the process of pathological (hyper- or hypo-) innervation in order to improve quality of life in patients with heart disease and decrease the incidence of sudden cardiac death.

## Abbreviations

The following abbreviations are used in this manuscript:

AV	Atrioventricular
BMP	Bone morphogenetic protein
cANS	Cardiac autonomic nervous system
CGRP	Calcitonin gene-related peptide
ChAT	Choline acetyltransferase
CNS	Central nervous system
DBH	Dopamine-β-hydroxylase
EMT	Epithelial-to-mesenchymal transition
EPDC	Epicardium-derived cell
GDNF	Glial cell line-derived neurotrophic factor
GFRα	α-GDNF family receptors
HAND2	Heart and neural crest derivatives expressed 2
HH	Hamburger and Hamilton stage
ICA	Intrinsic cardiac adrenergic
MASH	Mammalian achaete-scute homolog
MI	Myocardial infarction
MMP	Matrix metalloproteinase
NCC	Neural crest cells
NGF	Nerve growth factor
NRP	Neuropilin
PEO	Proepicardial organ
PHOX	Paired-like homeobox
PNMT	Phenylethanolamine-N-methyltransferase
PNS	Peripheral nervous system
SA	Sinoatrial
SEMASIF	Semaphorinsmall intensely fluorescent
SMC	Smooth muscle cell
TH	Tyrosine hydroxylase
VAChT	Vesicular acetylcholine transporter
VSMC	Vascular smooth muscle cell
WT1	Wilms’ tumor 1
